# Exosomes Derived From miR-212-5p Overexpressed Human Synovial Mesenchymal Stem Cells Suppress Chondrocyte Degeneration and Inflammation by Targeting ELF3

**DOI:** 10.3389/fbioe.2022.816209

**Published:** 2022-02-24

**Authors:** Tianlei Zheng, Yan Li, Xiaozai Zhang, Jia Xu, Ming Luo

**Affiliations:** ^1^ Department of Orthopaedics, Xijing Hospital, The Fourth Military Medical University, Xi’an, China; ^2^ Department of Orthopaedics, The First Affiliated Hospital of Xi’an Jiaotong University, Xi’an, China; ^3^ Tangdu Hospital, The Fourth Military Medical University, Xi’an, China

**Keywords:** miR-212-5p, synovial mesenchymal stem cells, ELF3, chondrocyte, SMSCs, osteoarthritis

## Abstract

Excessive chondrocyte degeneration and inflammation are the pathological features of osteoarthritis (OA), and altered miR-212-5p may contribute to meniscus and cartilage degeneration. Whether exosomes derived from miR-212-5p overexpressed synovial mesenchymal stem cells (SMSC-212-5p-Exos) could be utilized to treat degenerative chondrocytes is investigated in this study. Down-regulated miR-212-5p and up-regulated E74 Like ETS Transcription Factor 3 (ELF3) expression were detected in OA synovial tissues, which showed a negative correlation (*r* = −0.55, *p* = 0.002). miR-212-5p directly targeted ELF3 and regulated the relative expression of *ELF3* in SMSCs as indicated by luciferase reporter assay and RT-PCR. The relative expression of ELF3, chondrocyte degeneration-related molecules, matrix metalloproteinase, and inflammatory molecules were detected in chondrocytes stimulated with interleukin (IL)-1β or co-incubated with SMSC-212-5p-Exos or SMSCs-derived exosomes (SMSC-Exos). IL-1β induced up-regulation of ELF3, down-regulation of degeneration molecules (Collagen II, Aggrecan, and Sox9), up-regulation of matrix metalloproteinase (MMP-1, MMP-3, and MMP-13), and up-regulation of inflammatory molecules (IL-6, MCP-1, TNF-α, COX-2, and iNOS) could be inhibited by SMSC-212-5p-Exos or SMSC-Exos administration. When compared with the SMSC-Exos, SMSC-212-5p-Exos showed more treatment benefits. All of these indicate that SMSC-212-5p-Exos could suppress chondrocyte degeneration and inflammation by targeting ELF3, which can be considered as a disease-modifying strategy.

## Introduction

Osteoarthritis (OA) is a disease that comprises progressive cartilage degenerative and synovial membrane inflammation, having an increased frequency of osteophyte formation and subchondral bone sclerosis in the aging population (Hunter and Bierma-Zeinstra, 2019; Katz et al., 2021; Sharma, 2021). No curative treatment has been applied in the clinic, and joint replacement remains the most commonly applied and effective therapy for the disability. Chondrocyte degeneration and inflammation are the principal cause of OA, for chondrocytes are the only residents in the avascular articular cartilage to maintain the specialized structure. It is also reported that chondrocytes size could be potentially used to assess disease progression (Gratal et al., 2019). Therefore, the molecular mechanism underlying chondrocyte degeneration and inflammation is vital for OA treatment.

Exosomes are small size (30–100 nm), single-membrane organelles released by various cells to shunt loading bioactive molecules to target cells (Kalluri and LeBleu, 2020; O'Brien et al., 2020). Research has shown that exosomes derived from synovial mesenchymal stem cells (SMSC-Exos) have the potential to palliate the severity of interleukin (IL)-1β induced osteoarthritis (Wang et al., 2020; Qiu et al., 2021). Further research is urgent to confirm the effectiveness and feasibility of exosome-based therapy.

MicroRNAs (miRNAs) can post-transcriptionally regulate OA-associated genes in chondrocytes (Oliviero et al., 2019; Huang et al., 2021). Exosomal miRNAs differ significantly from those of the parent cell, and following uptake, exosomal miRNAs can mediate target gene repression and reprogramme the cellular response (Heidarzadeh et al., 2021; Moghadasi et al., 2021). In rheumatoid arthritis, miR-212-3p can reduce proliferation and promote apoptosis of fibroblast-like synoviocytes (Liu et al., 2018). On the other hand, altered miR-212-5p expression can lead to the meniscus and cartilage degenerative process in OA (Jiang et al., 2021). All of these indicate the treatment benefit of miR-212-5p delivery in OA and whether exosomes derived from miR-212-5p overexpressed SMSCs (SMSC-212-5p-Exos) could be utilized to treat OA is investigated in this study.

## Methods and Materials

### Patient Enrollment

Based on the criteria of the American College of Rheumatology, thirty OA patients (18 females and 12 males; 50–74 years old; mean age of 62.3 ± 6.4 years) underwent total knee arthroplasty were enrolled. Meanwhile, the synovial tissues were collected from OA patients and 20 donors with accidental deaths (excluding those with OA-related diseases) or normal knee-joint synovium who were subjected to lower limb amputation after acute trauma or open reduction and internal fixation after fracturing of the tibial plateau (13 females and 7 males; 50–75 years old; mean age of 61.87 ± 7.44 years). The study was approved by the Xijing Hospital, and informed written consent was derived from each participant or close relatives.

### Synovial Mesenchymal Stem Cells Differentiation and Transfection

Synovial membrane specimens were digested with 0.2% type I collagenase (Thermo Fisher) at 37°C overnight, which were further collected by centrifugation and seeded in a high-glucose DMEM medium (Thermo Fisher, Waltham, MA, United States) supplemented with 10% FBS for 4 days to allow cell attachment. The medium was refreshed every 3 days, and at day 14, SMSCs were obtained. After blocking with human BD Fc Block™, SMSCs were stained with the following antibodies (Becton Dickinson): anti-CD34, anti-CD44, anti-CD45, anti-Sca-1, and anti-CD105 antibodies to confirm the phenotype using Guava^®^ easyCyte™ flow cytometer (Merck-Millipore, Billerica, MA, United States). At passage 3, SMSCs were switched to osteogenic differentiation medium (Sigma-Aldrich, St. Louis, MO, United States) for 2 weeks or StemPro Adipogenesis Differentiation Kit (Gibco) for 4 weeks. The miR-212-5p mimic or mimic-negative control (NC) (Sigma-Aldrich) were transfected with Lipofectamine^®^ 3,000 (Thermo Fisher) at the concentration of 100 nM according to the manufacturer’s instructions. The following sequences were used: miR-212-5p mimic (sense, 5′-ACC​UUG​GCU​CUA​GAC​UGC​UUA​CU-3'; and antisense, 5′-UAA​GCA​GUC​UAG​AGC​CAA​GGU​UU-3′), mimic-negative control miRNA (sense, 5′-UUC​UCC​GAA​CGU​GUC​ACG​UTT-3'; and antisense, 5′-ACG​UGA​CAC​GUU​CGG​AGA​ATT-3′).

### Exosomes Isolation and Characterization

Conditioned medium of SMSCs or miR-212-5p overexpressing SMSCs was filtered with 0.22 μM filters (Merck-Millipore) and centrifuged (4,000×g) to concentrate the volume into approximately 200 μL, which was further ultracentrifuged (100,000×g, 1 h, 4°C) to obtain the exosomal particles. A transmission electron microscope (TEM) was utilized to observe the morphology of exosomes, and the size and distribution of exosomes were measured using dynamic light scattering (DLS) analysis.

### Collection and Culture of Primary Chondrocyte

Primary chondrocytes were detached from cartilages dissected from the subchondral bone with 0.25 mg/ml collagenase P and 4 mg/ml protease, and the isolated chondrocytes (1×10^7^) were seeded into the 6-well plates 1 day before treatment. When cell confluence reached 80%, 2 µg exosomes (EXO miR-NC and EXO-miR-212-5p) were introduced into the chondrocytes culture medium supplemented with 10 ng/ml IL-1β. Chondrocytes treated with PBS were regarded as the blank controls. After 48 h of treatment, cells were collected for subsequent use.

### RNA Isolation and Quantitation

TRIzol (Invitrogen) was utilized to extract total RNA from chondrocytes or SMSCs. Total Exosome RNA & Protein Isolation Kit (Invitrogen, Waltham, MA, United States) was used to extract RNA and protein from exosomes for further analysis. TaqMan™ Advanced miRNA cDNA Synthesis Kit (Thermofisher) was utilized to reverse-transcript miRNA into cDNA, and RNU6B was utilized as an internal reference. Transscript^®^ All-in-One-First-Strand cDNA synthesis supermix was utilized to reverse-transcript mRNA into cDNA, and GAPDH was utilized as an internal reference. SYBR Green Master Mix (Roche, Penzberg, Upper Bavaria, Germany) was applied to detect the amplification. The primers were listed as following: ELF3, forward 5′- CAT​GAC​CTA​CGA​GAA​GCT​GAG​C-3′, reverse 5′- GAC​TCT​GGA​GAA​CCT​CTT​CCT​C-3’; miR-212-5p, forward 5′- CAG​TCT​CCA​GTC​ACG​G-3′, reverse 5′- GAA​CAT​GTC​TGC​GTA​TCT​C-3’; Collagen II (COL2A1), forward 5′- CCT​GGC​AAA​GAT​GGT​GAG​ACA​G-3′, reverse 5′- CCT​GGT​TTT​CCA​CCT​TCA​CCT​G-3’; Aggrecan, forward 5′-CAG​GCT​ATG​AGC​AGT​GTG​ATG​C-3′, reverse 5′- GCT​GCT​GTC​TTT​GTC​ACC​CAC​A-3’; Sox9, forward 5′- AGG​AAG​CTC​GCG​GAC​CAG​TAC-3′, reverse 5′- GGT​GGT​CCT​TCT​TGT​GCT​GCA​C-3’; MMP-1, forward 5′- ATG​AAG​CAG​CCC​AGA​TGT​GGA​G -3′, 5′- TGG​TCC​ACA​TCT​GCT​CTT​GGC​A -3’; MMP-3, forward 5′- CAC​TCA​CAG​ACC​TGA​CTC​GGT​T-3′, reverse 5′- AAG​CAG​GAT​CAC​AGT​TGG​CTG​G-3’; MMP-13, forward 5′- CCT​TGA​TGC​CAT​TAC​CAG​TCT​CC-3′, reverse 5′- AAA​CAG​CTC​CGC​ATC​AAC​CTG​C-3’; IL-6, forward 5′ AGA​CAG​CCA​CTC​ACC​TCT​TCA​G-3′, reverse 5′- TTC​TGC​CAG​TGC​CTC​TTT​GCT​G-3; COX-2, forward 5′- CGG​TGA​AAC​TCT​GGC​TAG​ACA​G-3′, reverse 5′- GCA​AAC​CGT​AGA​TGC​TCA​GGG​A-3’; iNOS, forward 5′- GCT​CTA​CAC​CTC​CAA​TGT​GAC​C-3′, reverse 5′- CTG​CCG​AGA​TTT​GAG​CCT​CAT​G-3’; GAPDH, forward 5′- CTG​TGC​CGT​TGA​ATT​TGC​CG-3′, forward 5′- CGG​GTT​CCT​ATA​AAT​ACG​GAC​TG-3’.

### Luciferase Assay

The QuikChange Lightning Site-Directed Mutagenesis kit (Agilent, Santa Clara, CA, United States) was utilized to mutate the binding site of ELF3 with miR-212-5p. 3′-UTR fragment of ELF3 mRNA (mutant or wide type) was subcloned into pGL3 luciferase vector (Promega, Madison, WI, United States), which was further co-transfected with miR-212-5p mimic or mimic-NC into HEK-293T cells for 36 h with Lipofectamine 3,000. Subsequently, the luciferase activity was assayed with Luciferase Assay System (Promega).

### Immunoblot Assay

The cellular lysates were quantified using the BCA protein concentration kit (20201ES76, Yeasen Company, Shanghai, China), and 20 µg lysates were separated with 10% SDS-PAGE electrophoresis and transferred to PVDF membranes, which were further blocked in Tris-buffered saline with Tween 20 (TBST) containing 0.1% Tween 20 and 5% skimmed milk powder and followed by incubation with the primary antibodies (Santa Cruz, Dallas, TX, United States) specific for CD63, CD9, Alix, ELF3, and GAPDH, and then incubated in peroxidase-conjugated secondary antibody (Sigma-Aldrich) at room temperature for 1 h. The dilution used for the primary antibodies against CD63, CD9, Alix, and ELF3 was 1:1,000, the dilution used for the primary antibody against GAPDH was 1:2000, and the dilution used for the second antibody was 1:2000. The signal was developed with an ECL system (GE Healthcare), and the relative intensity was normalized with GAPDH expression with NIH-Image J1.51p 22.

### Elisa

According to the manufacturer’s instructions, the concentrations of IL-6, MCP-1, MMP-3, MMP-13, and TNF-α were detected with commercial ELISA kits (eBiosciences, San Diego, CA, United States). Separated stock standard solutions and samples were measured with a SpectraMax M5 microplate reader at a wavelength of 450 nm.

### Statistical Analysis

Statistical analysis were performed with Graph-Pad Prism 6.0. Data were presented as means ± SD. The Mann-Whitney test was performed to determine the statistical significance between any two groups, and Dunn’s multiple comparisons test was used to compare multiple groups. *p*-value < 0.05 was considered statistically significant.

## Results

### Down-Regulated miR-212-5p and Up-Regulated ELF3 Expression in OA Synovial Tissues

**FIGURE 1:**
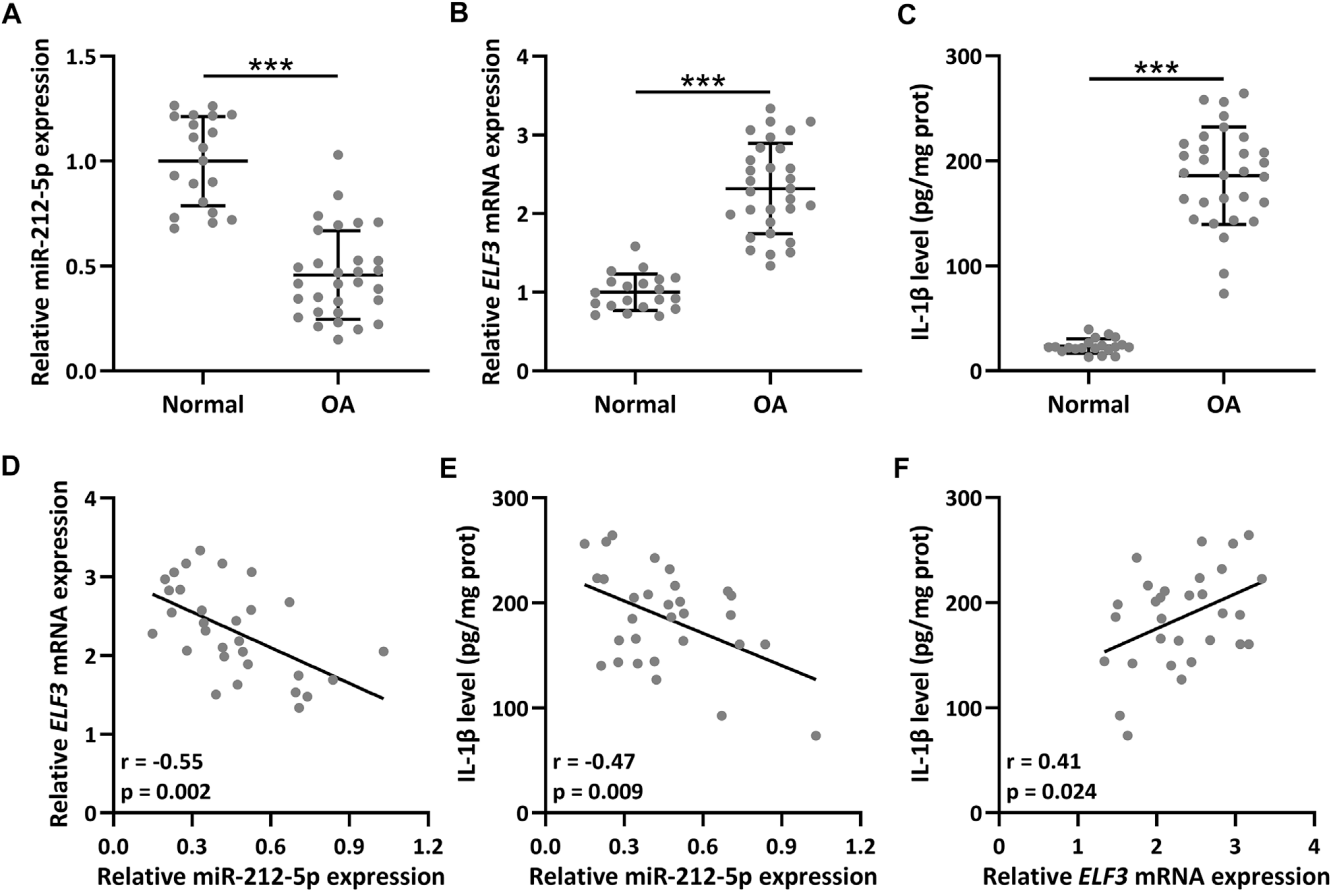
The expression of ELF3 and miR-212-5p in OA (*n* = 30) and normal synovial tissues (*n* = 20). qRT-PCR was utilized to detect the relative expressions of miR-212-5p **(A)** and ELF3 **(B)**. ELISA was used to measure the IL-1β level **(C)**. Pearson correlation coefficient of miR-212-5p and ELF3 mRNA **(D)**, miR-212-5p and IL-1β level **(E)**, ELF3 mRNA and IL-1β level **(F)** in OA synovial tissues. *n* = 30. Mean ± SD. ***p < 0.001.

The relative miR-212-5p expression in OA synovial tissues was down-regulated compared with healthy control (Figure 1A, *p* < 0.001), and up-regulated *ELF3* mRNA expression (Figure 1B, *p* < 0.001) and increased protein expression of IL-1β (Figure 1C, *p* < 0.001) were also observed. The expression correlation analysis indicated that both the correlation between *ELF3* and miR-212-5p (Figure 1D, *r* = −0.55, *p* = 0.002) and the correlation between IL-1β and miR-212-5p (Figure 1E, *r* = −0.47, *p* = 0.009) were significantly negatively correlated. As expected, the correlation between *ELF3* and IL-1β was positively correlated (Figure 1F, *r* = 0.41, *p* = 0.024).

### SMSC-Derived Exosomes Isolation and Confirmation

**FIGURE 2:**
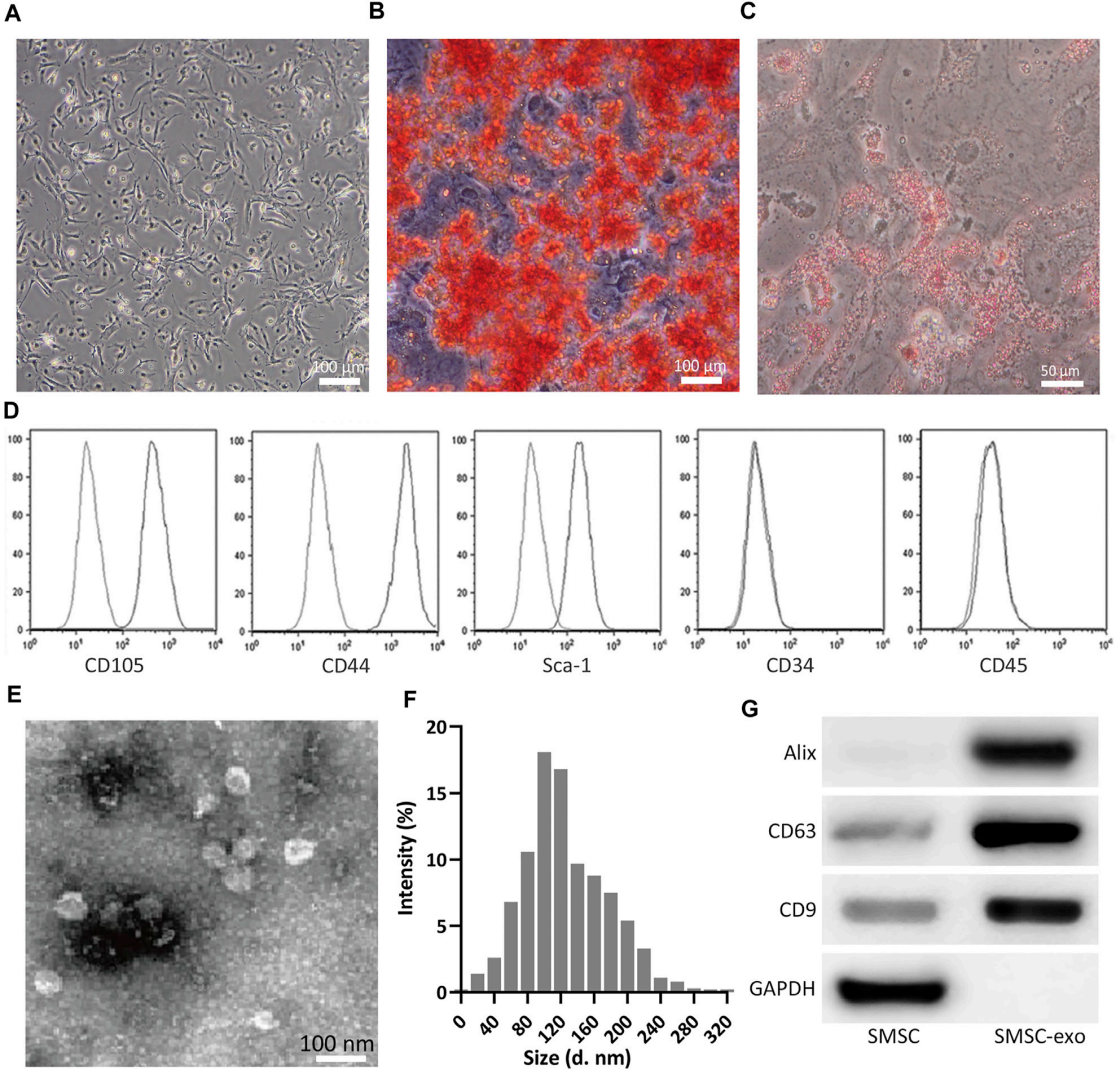
Isolation of SMSCs and SMSC-derived exosomes (SMSC-Exos). **(A)** Observation of SMSCs morphology at passage 3. **(B)** After 14 days of osteogenic differentiation, matrix mineralization of SMSCs was stained with Alizarin Red S. **(C)** after 14 days of adipogenesis induction, SMSCs were stained with Oil Red O. **(D)** Fluorescence Activated Cell Sorting was performed on SMSCs for CD105, CD44, Sca-1, CD34, and CD45 at passage 3. **(E)** Identification of exosomes by TEM. **(F)** Nanoparticle Tracking Analysis of the isolated exosome. **(G)** Detection of CD9, CD63, and Alix expression by Western blot analysis between SMSCs and SMSC-derived exosomes (SMSC-Exos).

Spindle-like SMSCs were observed at passage 3 (P3) (Figure 2A), which could be induced into osteogenic cells confirmed by Alizarin Red S staining of calcium mineral deposits (Figure 2B) and induced into adipogenic cells confirmed by Oil Red O staining of small cytoplasmic lipid droplets (Figure 2C). Flow cytometry analysis confirmed the phenotype of SMSCs, which were positive for CD105, CD44, and SCA-1, and negative for CD34 and CD45 (Figure 2D). TEM showed the single membrane structure of SMSC-Exos (Figure 2E), and dynamic light scattering assayed that most exosomes ranged from 30 to 150 nm in size (Figure 2F). The relative protein expression of exosome markers (CD63, CD9, and Alix) was significantly up-regulated as detected by Western blot (Figure 2G). All these results confirmed the success of SMSC-Exos isolation.

### miR-212-5p Directly Targets ELF3 in SMSCs

**FIGURE 3:**
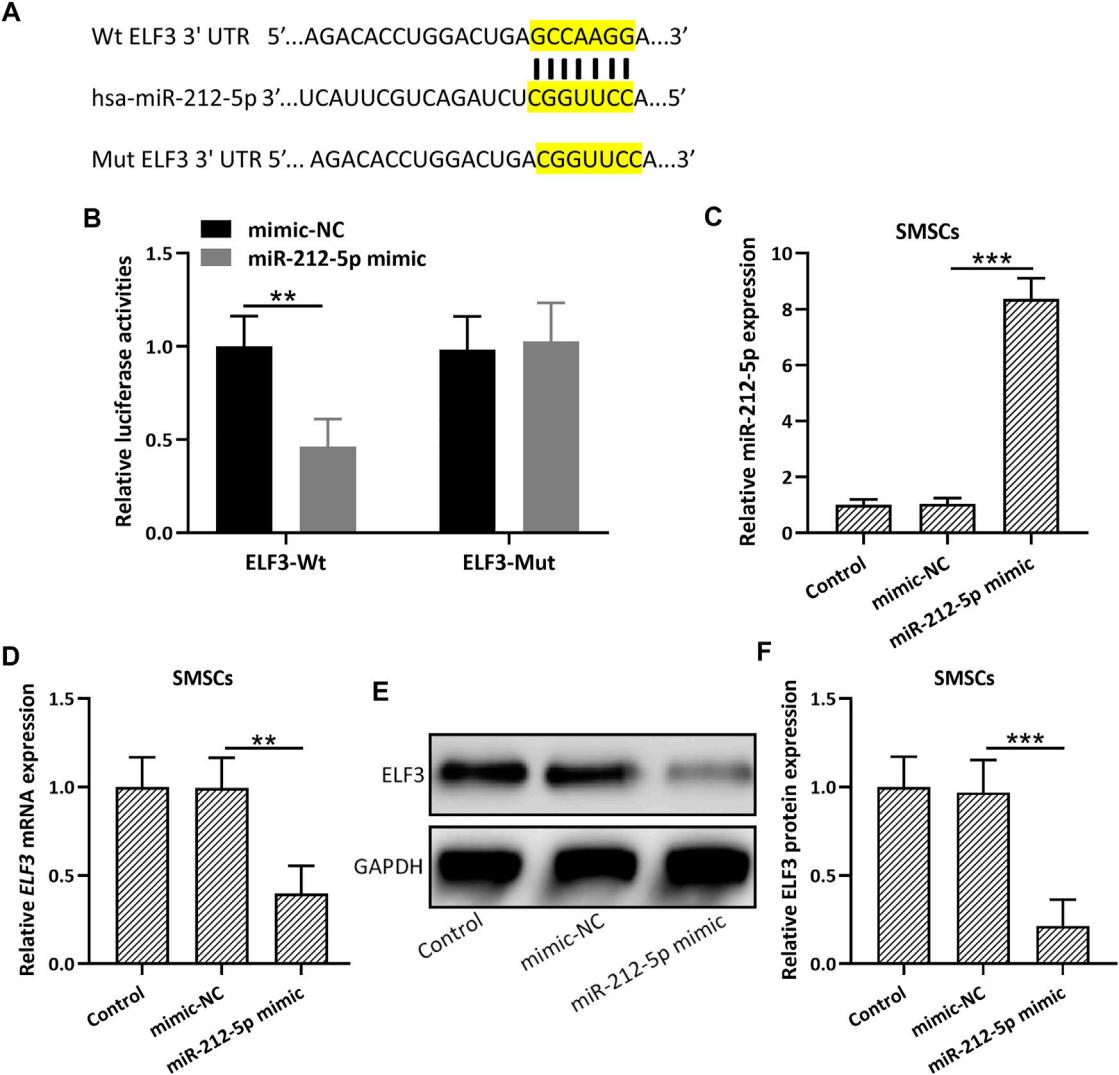
miR-212-5p targeted ELF3 and regulated the expressions of ELF3 in SMSCs. **(A)** The predicted binding sequences of miR-212-5p with ELF3 mRNA were shown. **(B)** HEK-293T cells were co-transfected with luciferase reporters containing ELF3 3′-UTR (wide type or mutant) and miR-212-5p mimics or negative control. After 48 h of incubation, the relative luciferase activity was measured. *n* = 3. Mean ± SD. **p < 0.01, Two-way ANOVA followed Turkey’s multiple comparisons test. **(C)** qRT-PCR was used to assay the expressions of miR-212-5p in SMSCs and miR-212-5p overexpressed SMSCs. **(D–F)**, SMSCs were transfected with miR-212-5p mimics or negative control for 48 h qRT-PCR and Western blotting were used to analyze the mRNA and proteins expressions of ELF3. *N* = 3 for each group. Mean ± SD. **p < 0.01, ***p < 0.001, One-way ANOVA followed Dunn’s multiple comparisons test.

TargetScan (Agarwal et al., 2015) and miRBase (Ambros et al., 2003) database were utilized to predict the possible targets of miR-212-5p. ELF3 was among the top 10 molecules that could be binding with miR-212-5p. 3′-UTR region of ELF3, containing the predicted miR-212-5p binding sequences (Figure 3A), was cloned into the luciferase vector. MiR-212-5p overexpression could significantly decrease the luciferase activity of ELF3 (Figure 3B). In addition, up-regulation of miR-212-5p was observed in miR-212-5p overexpressed SMSCs (Figure 3C), which could diminish the relative mRNA (Figure 3D) and protein (Figures 3E,F) expression of ELF3. These results indicated that miR-212-5p directly targeted ELF3 and regulated the expression of ELF3 in SMSCs.

### Exosomes Derived From miR-212-5p Overexpressed SMSCs (SMSC-212-5p-Exos) Inhibit IL-1β Induced ELF3 Expression in Chondrocytes

**FIGURE 4:**
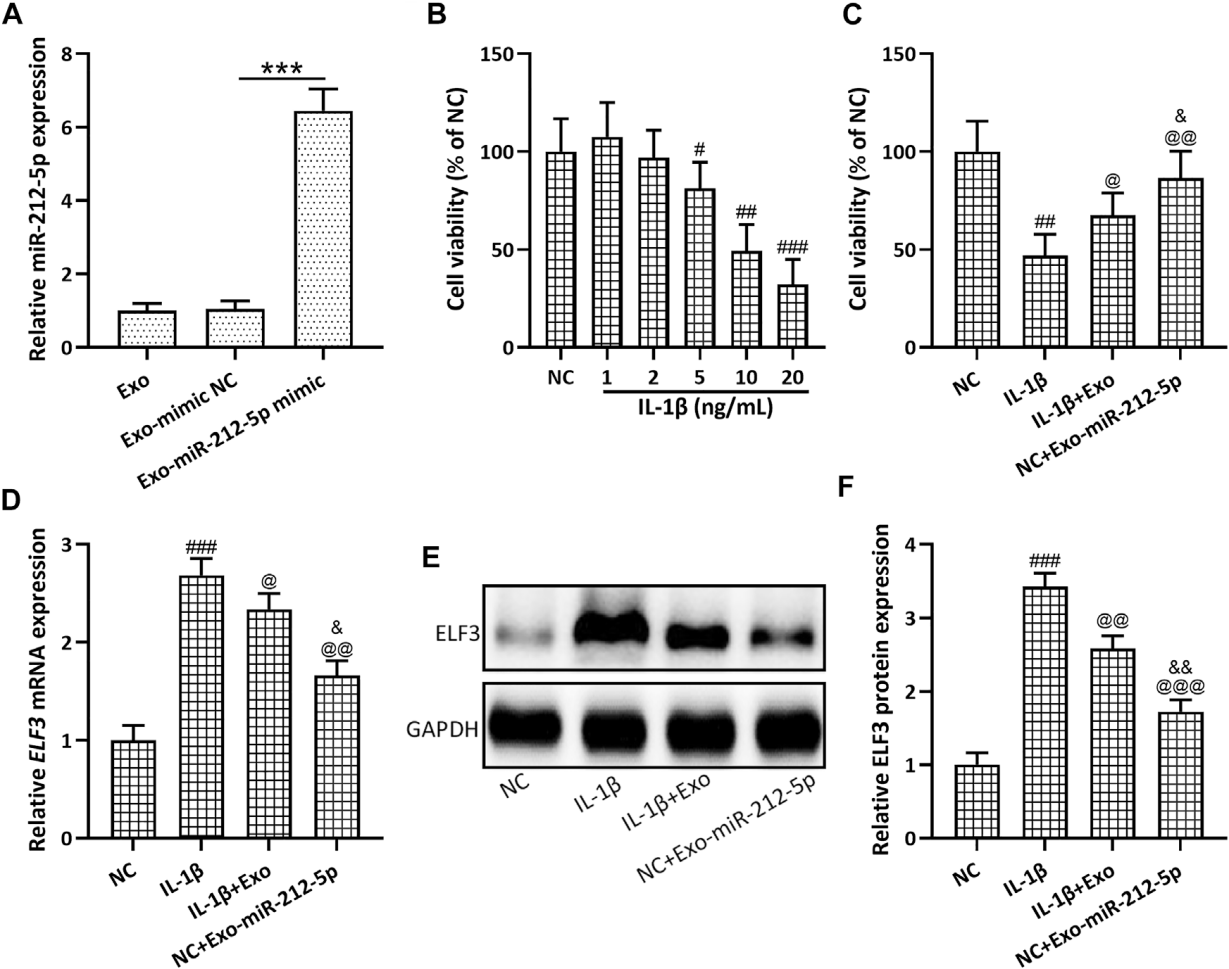
Exosomes derived from miR-212-5p overexpressed SMSCs (SMSC-212-5p-Exos) inhibited up-regulated ELF3 expression in chondrocytes induced by IL-1β. **(A)** qRT-PCR was utilized to analyze the relative expressions of miR-212-5p from SMSC-Exos or SMSC-212-5p-Exos. **(B)** Cell viability of chondrocytes after individual treatment with different concentrations of IL-1β for 48 h. **(C)** Cell viability of chondrocytes after co-treatment with IL-1β and SMSC-Exos, or SMSC-212-5p-Exos for 48 h. **(D–F)** IL-1β induced chondrocytes were treated with SMSC-Exos or SMSC-212-5p-Exos for 48 h qRT-PCR and western blotting were used to analyze the mRNA and proteins expressions of ELF3. *n* = 6 for each group in cell viability analysis, *n* = 3 for each group in qRT-PCR and western blotting tests. #*p* < 0.05, ##*p* < 0.01, ###*p* < 0.001 compared to NC. @*p* < 0.05, @@*p* < 0.01, @@@*p* < 0.001 compared to IL-1β. &*p* < 0.05, &&*p* < 0.01 compared to IL-1β+Exo. One-way ANOVA followed Dunn’s multiple comparisons test.

Up-regulated miR-212-5p expression could be detected in SMSC-212-5p-Exos (Figure 4A), indicating that SMSCs exosomes can carry miR-212-5p to target cells. SMSC-212-5p-Exos showed no difference in size and membrane structure when compared with SMSC-Exos (data not shown). Different concentrations of IL-1β were used to stimulate chondrocytes, and it was found that decreased cell viability was observed in accordance with the increased IL-1β concentration (Figure 4B). SMSC-Exos could reverse the diminished cell viability induced by IL-1β in chondrocytes, and SMSC-212-5p-Exos showed more treatment benefit when compared with SMSC-Exos (Figure 4C). On the other hand, IL-1β incubated chondrocytes showed up-regulated ELF3 expression in both transcription (Figure 4D) and protein levels (Figures 4E,F), which can be reversed by the co-incubation with SMSC-Exos or SMSC-212-5p-Exos, while SMSC-212-5p-Exos showed the better treatment benefit. All of these results indicated that exosomal miR-212-5p inhibited up-regulated ELF3 expression in chondrocytes induced by IL-1β.

### SMSC-212-5p-Exos Attenuate IL-1β Induced Chondrocyte Degeneration and Degradation

**FIGURE 5:**
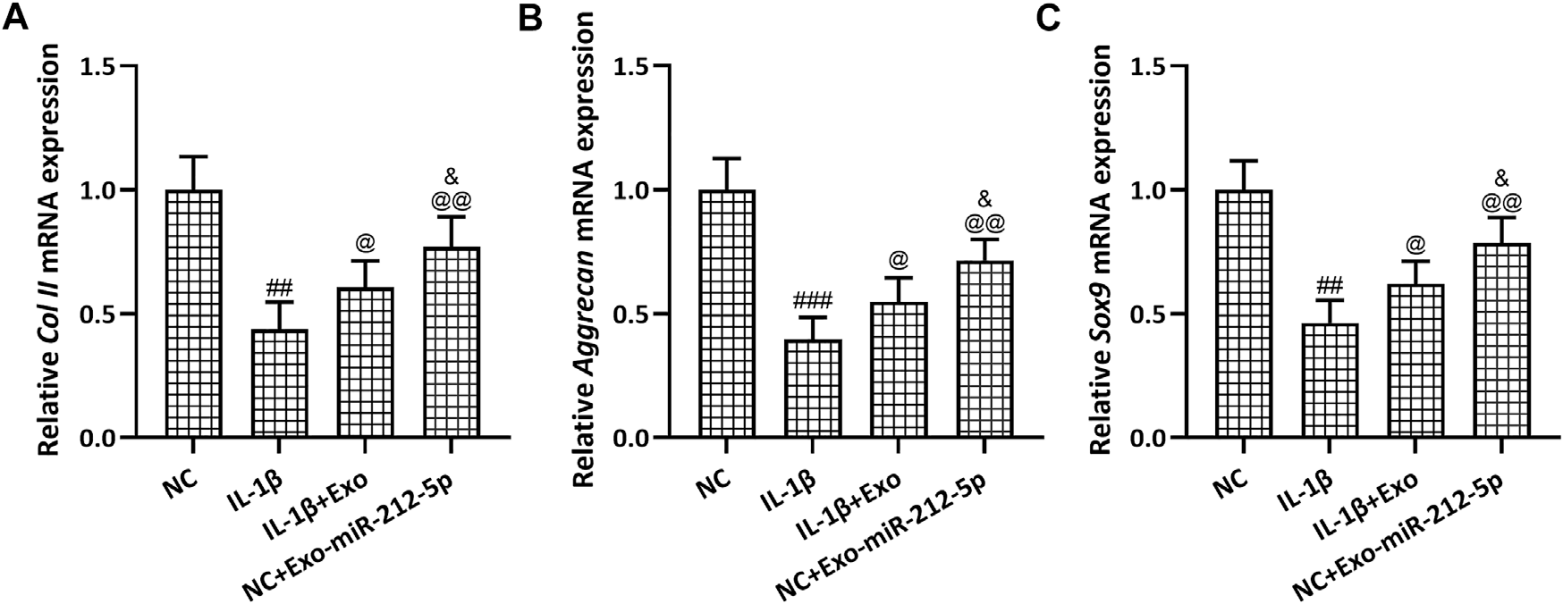
SMSC-212-5p-Exos attenuated IL-1β induced chondrocyte degeneration. IL-1β induced chondrocytes were treated with SMSC-Exos or SMSC-212-5p-Exos for 48 h qRT-PCR was utilized to analyze the mRNA expressions of Collagen II **(A)**, Aggrecan **(B)** and Sox9 **(C)**. *n* = 3 for each group. ##*p* < 0.01, ###*p* < 0.001 compared to NC. @*p* < 0.05, @@*p* < 0.01 compared to IL-1β. &*p* < 0.05 compared to IL-1β+Exo. One-way ANOVA followed Dunn’s multiple comparisons test.

**FIGURE 6:**
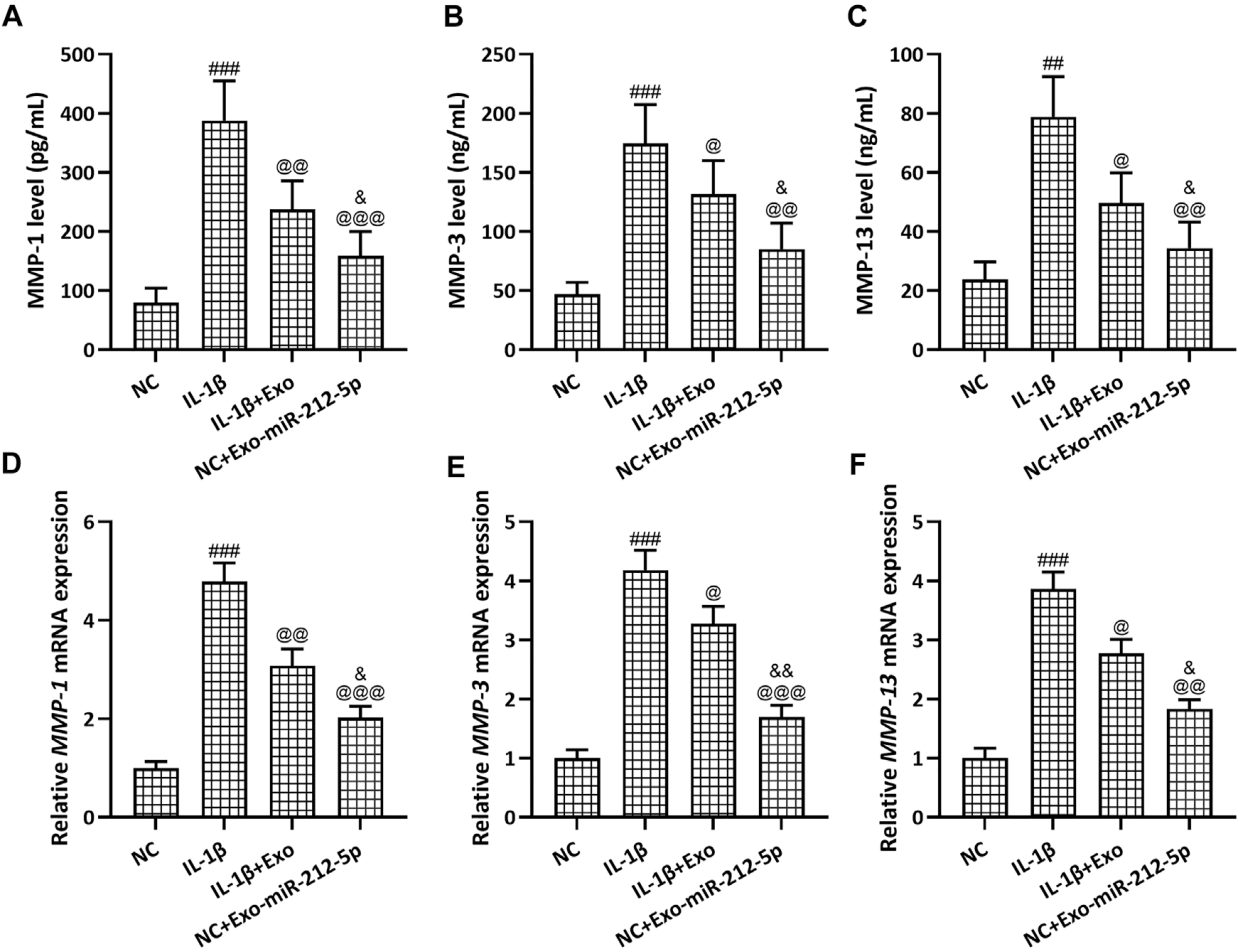
SMSC-212-5p-Exos attenuated IL-1β induced MMP-1, MMP-3, and MMP-13 expression in chondrocytes. IL-1β induced chondrocytes were treated with SMSC-Exos or SMSC-212-5p-Exos for 48 h. MMP-1, MMP-3, and MMP-13 production in cellular supernatant were measured by ELISA **(A–C)**. mRNA expression of MMP-1, MMP-3, and MMP-13 from cell lysis were tested by qRT-PCR **(D–F)**. *n* = 6 for each group in ELISA analysis, *n* = 3 for each group in qRT-PCR test. ##*p* < 0.01, ###*p* < 0.001 compared to NC. @*p* < 0.05, @@*p* < 0.01, @@@*p* < 0.001 compared to IL-1β. &*p* < 0.05, &&*p* < 0.01 compared to IL-1β+Exo. One-way ANOVA followed Dunn’s multiple comparisons test.

The relative mRNA expression of Collagen II (Figure 5A), Aggrecan (Figure 5B), and SOX9 (Figure 5C) in chondrocytes was decreased after the stimulation with IL-1β, while SMSC-Exos, especially SMSC-212-5p-Exos, could reverse such reduced expression of chondrocyte degeneration related molecules. The relative content of MMP-1 (Figure 6A), MMP-3 (Figure 6B), and MMP-13 (Figure 6C) in the culture supernatant of chondrocytes was increased after the stimulation with IL-1β, while SMSC-Exos or SMSC-212-5p-Exos could inhibit the secretion of the relevant molecules. On the other hand, the relative expression of MMP-1 (Figure 6D), MMP-3 (Figure 6E), and MMP-13 (Figure 6F) in chondrocytes was increased after the treatment with IL-1β, while SMSC-Exos or SMSC-212-5p-Exos could reverse such increased expression. When compared with SMSC-Exos treatment, SMSC-212-5p-Exos treatment could show more treatment benefit. These results demonstrated that exosomal miR-212-5p could attenuate IL-1β induced chondrocyte degeneration and degradation.

### SMSC-212-5p-Exos Attenuate IL-1β Induced Inflammatory Responses in Chondrocytes

**FIGURE 7:**
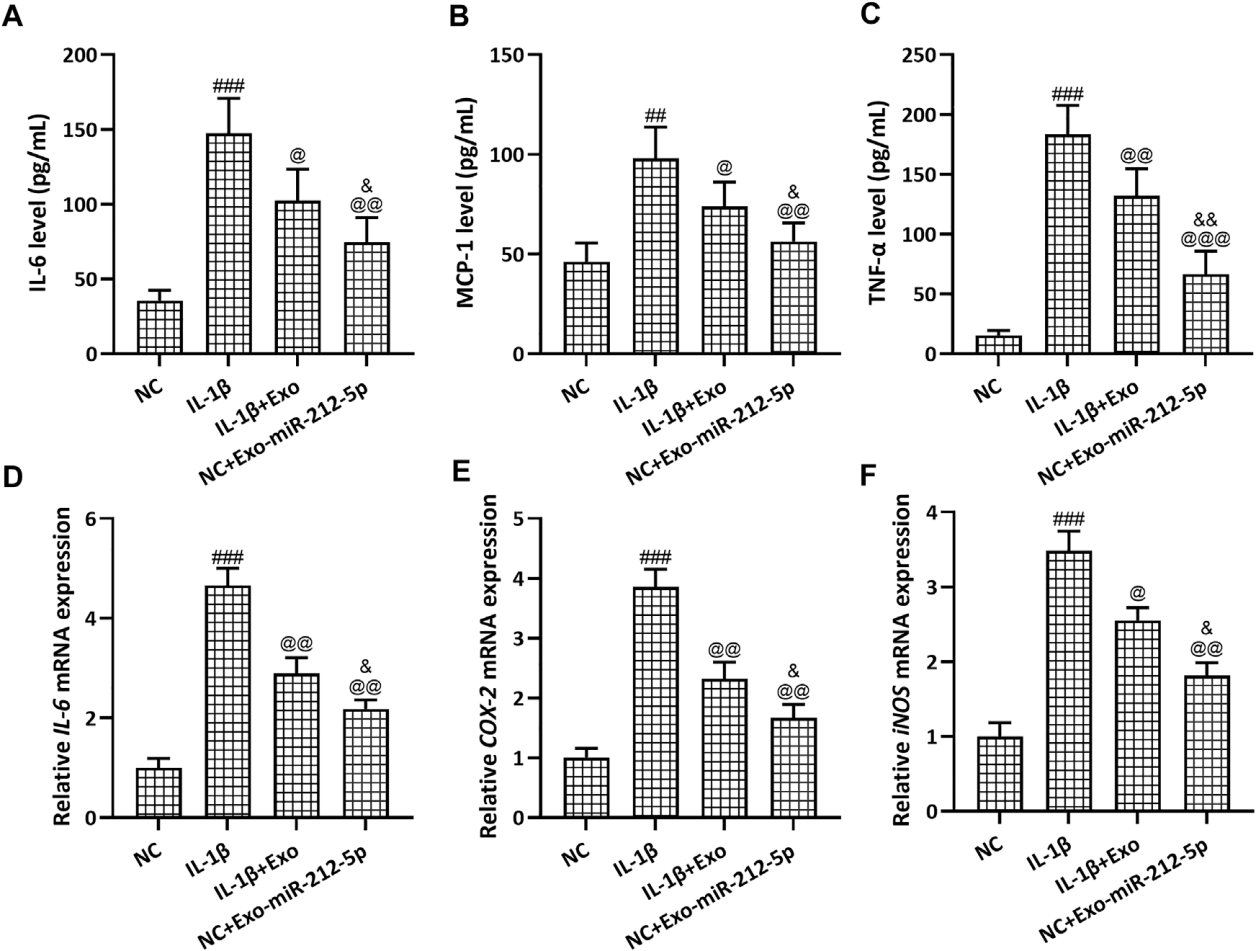
SMSC-212-5p-Exos attenuated IL-1β induced inflammatory responses in chondrocytes. IL-1β induced chondrocytes were treated with SMSC-Exos or SMSC-212-5p-Exos for 48 h. IL-6, MCP-1, and TNF-α production in cellular supernatant were measured by ELISA **(A–C)**. mRNA levels of *IL-6*, *COX-2*, and *iNOS* from cell lysis were tested by qRT-PCR **(D–F)**. *n* = 6 for each group in ELISA analysis, *n* = 3 for each group in qRT-PCR test. ##*p* < 0.01, ###*p* < 0.001 compared to NC. @*p* < 0.05, @@*p* < 0.01, @@@*p* < 0.001 compared to IL-1β. &*p* < 0.05, &&*p* < 0.01 compared to IL-1β+Exo. One-way ANOVA followed Dunn’s multiple comparisons test.

The relative content of IL-6 (Figure 7A), MCP1 (Figure 7B), and TNFα (Figure 7C) in the culture supernatant of chondrocytes was increased after the treatment with IL-1β, while SMSC-Exos or SMSC-212-5p-Exos could reverse such increased expression. On the other hand, the relative mRNA expression of IL-6 (Figure 7D), COX-2 (Figure 7E), and iNOS (Figure 7F) in chondrocytes was increased after the treatment with IL-1β, while SMSC-Exos or SMSC-212-5p-Exos could reverse such increased expression. It was worth noting that when compared with SMSC-Exos treatment, SMSC-212-5p-Exos treatment could decrease the levels of inflammatory molecules.

## Discussion

In addition to inflammation, dysregulation between the synthesis and degradation of extracellular matrix mainly mediated by type-II collagen, aggrecan, and matrix metalloproteinases contributes to the articular cartilage degradation and loss process (Martel-Pelletier et al., 2016). In this study, we utilize IL-1β induced chondrocytes to mimic the pathology of OA. We found that SMSC-Exos showed the enrichment of miR-212-5p, which could target chondrocytes and further inhibit the ELF3 mediated chondrocyte degeneration, degradation, and inflammation process. This study highlights the significance and the need for further development of SMSC-212-5p-Exos on the clinical therapy of OA.

miR-132/212 clusters are maintained on down-regulation in mesenchymal stem cells at four different stages of transforming growth factor-β3-induced chondrogenesis differentiation (Yang et al., 2011). On the other hand, miR-132/212 clusters regulate hematopoietic stem cell maintenance and survival with age by buffering forkhead box O-3 (FoxO3) expression (Mehta et al., 2015). While no research has been performed to decipher the role of miR-212-5p in SMSCs. In our study, miR-212-5p is identified to contribute to the regulation of chondrocytes mediated by SMSC-Exos or SMSC-212-5p-Exos. All of these results indicate the universal utilization of miR-212-5p based treatment.

As transcription factors belonging to the ETS family, ELF3 participates in autoimmune and tumor neogenesis (Jedlicka and Gutierrez-Hartmann, 2008; Luk et al., 2018). The previous study demonstrates that ELF3 could modulate type II collagen transcription by prohibiting SOX9-CBP/p300-driven histone acetyltransferase activity in chondrocytes (Otero et al., 2017). On the other hand, ELF3 co-localizes with MMP-13 protein to regulate the transcription activity in human osteoarthritic cartilage (Otero et al., 2012). In accordance with the up-regulated SOX9 and MMP-13 expression testified in our study, all of these results define a pro-catabolic role for ELF3 to regulate MMP-13 and SOX9 mediated cartilage remodeling and degradation. Elevated ELF3 expression in OA cartilage tissues can act as a mediator of inflammatory and catabolism to destruct cartilage, which indicates the potential target of ELF3 to alleviate OA (Conde et al., 2018).

ELF3 is identified to direct target miR-212 to suppress nasopharyngeal carcinoma cells proliferation (Kang et al., 2020). In this research, miR-212-5p could target ELF3 to alleviate IL-1β induced chondrocytes degradation and inflammation. There are some limitations that should be indicated here. Microenvironment can alter the composition of exosomes (Skuratovskaia et al., 2020), and *in vivo* models should be constructed to demonstrate the therapeutic benefit of SMSC-212-5p-Exos. Whether other molecules, except miR-212-5p, could contribute to the treatment advantage in SMSC-212-5p-Exos compared with SMSC-Exos need further detailed analysis.

Accumulating investigations have indicated that exosomes will pave a new therapeutic paradigm for osteoarthritis treatment. Our study testifies that SMSC-212-5p-Exos could alleviate IL-1β induced chondrocytes degradation, degradation, and inflammation process involved in OA. It is worth noting that only *in vitro* model is utilized in the study, and the OA *in vivo* model should be constructed to demonstrate the treatment benefit of SMSC-212-5p-Exos. All in all, this study indicates that SMSC-212-5p-Exos could target ELF3-mediated OA pathology in chondrocytes, which can be considered as a future treatment option.

## Data Availability

The raw data supporting the conclusion of this article will be made available by the authors, without undue reservation.
